# High‐pressure processing applied to sliced dry‐cured Iberian loin: Effect of category, company, and storage temperature

**DOI:** 10.1002/fsn3.3507

**Published:** 2023-06-16

**Authors:** Ana Isabel Carrapiso, David Tejerina, Susana García‐Torres, Antonia Trejo, Maria Jesus Martín Mateos, Jonathan Delgado‐Adámez, Jesús Javier García‐Parra, Maria Rosario Ramírez

**Affiliations:** ^1^ Tecnología de Alimentos, Escuela de Ingenierías Agrarias Universidad de Extremadura Badajoz Spain; ^2^ Technological Agri‐Food Institute (INTAEX)/La Orden, Center for Scientific and Technological Research of Extremadura (CICYTEX) Badajoz Spain

**Keywords:** between‐companies effect, dry‐cured Iberian loin, high‐pressure processing, loin category, sliced *Bellota* loin, storage temperature

## Abstract

The main purpose of this study was to evaluate the effect of high‐pressure processing (HPP) and storage temperature on the microbial counts, the instrumental color, and the oxidation stability of sliced dry‐cured Iberian loin from two categories and two leading companies. 600 MPa for 8 min was sufficient to decrease all the microbial counts without affecting the color and the oxidation status, the effect being modulated by the loin category and company, whose effect on those variables was marked. However, the subsequent 90‐day storage softened the initial effect of HPP on microorganisms and allowed a significant effect of HPP to develop on color and oxidation. In addition, the coliform counts were higher after storage at 20°C than at 4°C, suggesting that refrigeration may be needed during long‐term storage to ensure loin safety.

## Graphical abstract

Loin category and company had a marked effect and influenced the effect of HPP. Just applied, HPP lowered the microbial counts without affecting color and oxidation. Storage reduced the effect of HPP on the counts but raised it on color and oxidation. Storage did not decrease the coliform counts. Storage at 4°C resulted in lower coliform counts than at 20°C.

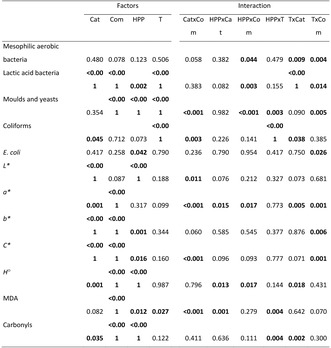

## 1 INTRODUCTION

Dry‐cured loin is an appreciated Iberian pork product with unique sensory traits, different from other Iberian products due to the use of seasonings. The entire Iberian loin has a considerably long shelf life. However, it is increasingly being sold sliced and vacuum packaged as a ready‐to‐eat product. This has arisen new concerns related to the quality loss favored by the slicing step, including discoloration and oxidation (Fuentes et al., 2010), and safety concerns due to the risk of microbial contamination, which may affect both the shelf life and loin safety.

High‐pressure processing (HPP) is a nonthermal technology effective at diminishing microbial counts and ensuring safety in Iberian dry‐cured meat products (Cava et al., 2020, 2021; Martillanes et al., 2021), with a generally modest detrimental effect (Carrapiso et al., 2021; Cava et al., 2009). Differences in the HPP‐induced damage have been found between several Iberian products (Cava et al., 2009, 2021), which implies that the damage may depend on the characteristics of each product. Nevertheless, scarce information is available on whether the initial loin characteristics influence the effect of HPP, the only factor researched so far being the intramuscular fat content (Fuentes et al., 2014).

However, there are more factors influencing loin characteristics and quality, such as the rearing system and breed. They are the basis of the official grading of Iberian loin, where the highest grade is “Bellota 100% Iberian” (only from pure Iberian pigs reared outdoors on acorns and grass), and the lowest is “Cebo” (at least 50% Iberian × Duroc pigs reared indoors on concentrate feedings) (Real Decreto 4/2014, 2014). Although several studies have reported marked differences among the loin categories, there is a lack of consistency on some parameters (e.g., oxidation) (Contador et al., 2021; Ramirez et al., 2021; Soto et al., 2008; Ventanas, Ventanas, et al., 2006). This suggests that additional factors might be involved in loin variability. One of them might be the specific manufacturing procedure applied by each company, including the added seasonings. So far, the effect of HPP has not been researched on the highest grade, nor has been the differences between companies.

Another critical factor for vacuum‐packaged sliced loin is the temperature during distribution, storage, and marketing, usually kept under 7°C. Recent studies have shown that storage at room temperature does not compromise safety in dry‐cured Cebo Iberian products but increases it instead when compared with refrigerated storage (Cava et al., 2020, 2021; Martillanes et al., 2021). However, those studies did not assess some microorganisms of concern, such as coliforms, or the effect on Bellota 100% Iberian loin. In addition, this economically convenient temperature might also increase oxidation and discoloration, the extent of them depending on the type of Iberian meat product (Cava et al., 2020, 2021; Martin et al., 2021). However, no information is available about the effect on Iberian loin from different categories.

Therefore, the main objective of this study was to evaluate the effect of HPP and storage temperature on the microbial counts, the instrumental color, and the oxidation stability of sliced dry‐cured Iberian loin (specifically two types of loin, both produced by two manufacturing companies).

## 2 MATERIALS AND METHODS

### 2.1 Samples

Two types of Iberian loin (the highest grade, called “Bellota 100% Iberian”, from Iberian pigs; and the lowest one, called “Cebo”, from 50% Iberian × Duroc pigs) sliced, vacuum packaged and with the compulsory black and white labels, respectively, were both purchased from two leading companies, taking them at random from the fresh packages produced for consumers. Each company used its usual procedures to produce both products, the labels declaring that the raw loins had been seasoned in a mixture of salt, paprika, potassium nitrate, sodium nitrite, sodium ascorbate, and either spices and dextrose (company A) or garlic, sucrose, lactose, milk protein, disodium dihydrogen diphosphate, and sodium polyphosphate (company B). Both companies applied similar processing conditions, common in the Iberian loin industry, which included refrigeration for 4–6 days (depending on weight), stuffing into collagen casings, and ripening to complete approx. 85 days (Bellota loins) or 70 days (Cebo loins). Then, they were peeled, sliced (approx. 1.5 mm), and vacuum packaged (approx. 100 g per package) according to each company's usual procedure, namely, in high‐density polypropylene (oxygen permeability ≤9.3 cm^3^ O_2_/m^2^/24 h at 4°C, company A) and PET‐PE (oxygen permeability ≤100 cm^3^/m^2^/24h at 23°C, 0% RH, company B) bags.

### 2.2 Experimental design

The packages were allotted to the Control and HPP (high‐pressure processing) groups and stored for 90 days at 4°C or 20°C. Five packages per batch were sampled before and after storage, with a total of 120 packages: 40 nonstored packages (5 packages × 2 HPP treatments (control vs. HPP) × 2 loin categories × 2 companies) and 80 packages stored for 90 days (5 packages × 2 storage temperatures × 2 HPP treatments × 2 loin categories × 2 companies).

### 2.3 High hydrostatic pressure and storage

A semi‐industrial high hydrostatic pressure unit (55 L of capacity, Hiperbaric Wave 6000/55) was used to pressurize the packages at 600 MPa for 8 min, sufficient to ensure inactivation of pathogens, such as *Listeria spp* (Cava et al., 2021; Ferreira et al., 2016). The initial water temperature inside the vessel was 10°C, and the time to reach 600 MPa was 230 s. HPP was applied as soon as the packages from each company arrived at the facility, all the packages from each company being treated in the same batch.

The packages were stored in the dark at 4°C ± 1°C or 20°C ± 2°C for 90 days.

### 2.4 Initial characterization

#### 2.4.1 Proximate analysis

The moisture, fat, and protein content were determined by drying the samples at 104°C until constant weight was achieved, gravimetrically after extraction with chloroform: methanol (2:1), and by the Kjeldahl method, respectively (Trejo et al., 2021).

#### 2.4.2 Fatty acid profile

The fatty acid profile was determined by obtaining the fatty acid methyl esters (FAMEs) using KOH in methanol and analyzing them using an Agilent Technologies 6890 GC equipped with a flame ionization detector and an Agilent DB‐23 (60 m × 0.25 mm ID × 0.25 μm) column. The injector and detector were maintained at 230°C, and the oven temperature rose from 185°C to 220°C (5°C min^−1^), with nitrogen as carrier gas (1.2 mL min^−1^). FAME identification was based on retention times. Quantification was performed by using tridecanoic acid as internal standard and the response factors were determined after injecting solutions with FAME standards and tridecanoate (Sigma). Results were expressed as required by the current regulation (Orden PRE/3844/2004, n.d.).

### 2.5 Microbiological analyses

Ten grams of loin was taken aseptically and homogenized with 90 mL of peptone water (Merck, 1.07043) in a laboratory blender (Stomacher® 400 Circulator). One milliliter of serial decimal dilutions in sterile peptone water was poured or spread onto total count and selective agar plates (Amaro‐Blanco et al., 2018) for most microorganisms and Chromocult agar (Merck) incubated at 37°C for 24–48 h for coliforms. The detection limit was 10 CFU g^−1^, except for *Staphylococcus aureus* (100 CFU g^−1^). The absence in 25 g of loin was evaluated for *Salmonella* and *L. monocytogenes*.

### 2.6 Instrumental color

The CIELAB instrumental color determinations were performed using a Minolta CM‐5 spectrophotometer (Minolta Camera, Osaka, Japan), with an illuminant/angle of D65/10^0^ and a measuring area of 30 mm. Each package was opened and left at room temperature (20°C ± 2°C) for approx. 30 min before the color measurements.

### 2.7 Oxidative status

Lipid oxidation was assessed using the thiobarbituric acid reactive substances (TBA‐RS) method (Sorensen & Jorgensen, 1996), using a standard curve of tetraethoxypropane.

Protein oxidation was evaluated by measuring the carbonyl groups formed during incubation with 2,4‐dinitrophenylhydrazine (DNPH) in 2 N HCl (Oliver et al., 1987).

### 2.8 Statistical analysis

A two‐way (loin category, company) ANOVA with interaction was performed on the data from the chemical characterization. For the other data before storage, a three‐way (loin category, company, HPP) ANOVA with interaction was applied. Finally, the data from the 90‐day stored samples were analyzed by using a four‐way (loin category, company, HPP, storage temperature) ANOVA with interaction. When the ANOVA showed a significant effect (*p* ≤ .05), the Tukey's test was performed to compare in pairs the sample groups.

## 3 RESULTS AND DISCUSSION

### 3.1 Effect of loin category and company before storage

The two types of Iberian loin (called Bellota 100% Iberian and Cebo) from the two leading companies differed initially in the moisture, protein, and intramuscular fat (IMF) content, three out of 12 fatty acids (Table 1), some microbial counts (mesophilic and lactic acid bacteria, and coliforms) and most instrumental color coordinates (Table 2). The “Bellota 100% Iberian” loin was manufactured from 100% Iberian pigs reared outdoors on acorns and grass, while “Cebo” loin was manufactured from at least 50% Iberian × Duroc pigs reared indoors on concentrate feedings. Differences between companies were larger than between loin categories: although the proximate composition was less influenced (only IMF was), four out of 12 fatty acids, most microbial counts (all except the mesophilic bacteria), three color coordinates (a*, C*, *H*°), and lipid and protein oxidation were significantly affected.

**TABLE 1:** Results* from the chemical characterization and fatty acid profile of vacuum‐packaged sliced loin.

	Bellota 100% Iberian		Cebo		*p*		
	Company A	Company B	Company A	Company B	Cat	Com	Cat × Com
Moisture (g 100 g^−1^)	39.7 ± 0.7^b^	37.3 ± 0.3^b^	44.3 ± 1.2^a^	44.0 ± 0.6^a^	**<.001**	.111	.231
Protein (g 100 g^−1^)	37.1 ± 1.0^ab^	40.1 ± 0.6^a^	36.2 ± 1.5^ab^	35.6 ± 1.1^b^	**.023**	.292	.118
Intramuscular fat (g 100 g^−1^)	14.5 ± 0.3^a^	11.3 ± 0.5^ab^	11.2 ± 1.3^ab^	8.9 ± 1.3^b^	**.011**	**.015**	.640
Fatty acids (%)**¶							
C12:0	0.1 ± 0.0	0.1 ± 0.0	0.1 ± 0.0	0.1 ± 0.0	.654	.085	.983
C14:0	1.4 ± 0.0	1.4 ± 0.1	1.4 ± 0.0	1.4 ± 0.1	.500	.375	.510
C16:0	26.1 ± 0.2	25.8 ± 0.1	25.7 ± 0.2	25.6 ± 0.1	.294	.479	.766
C16:1	3.6 ± 0.1^b^	4.4 ± 0.2^a^	4.1 ± 0.1^ab^	4.1 ± 0.1^ab^	.796	**.019**	**.014**
C17:0	0.2 ± 0.0	0.2 ± 0.0	0.2 ± 0.1	0.2 ± 0.0	.513	.708	.369
C17:1	0.2 ± 0.0	0.2 ± 0.0	0.2 ± 0.0	0.2 ± 0.0	.906	.213	.996
C18:0	12.3 ± 0.2	11.3 ± 0.5	12.1 ± 0.2	12.5 ± 0.2	.145	.346	**.033**
C18:1	49.5 ± 0.2	51.0 ± 1.0	51.0 ± 0.5	50.4 ± 0.1	.407	.405	.067
C18:2	5.2 ± 0.2^a^	4.2 ± 0.1^ab^	3.4 ± 0.1^b^	4.4 ± 0.2^ab^	**<.001**	.969	**<.001**
C18:3	1.4 ± 0.0^ab^	0.4 ± 0.0^b^	1.7 ± 0.0^a^	0.3 ± 0.0^b^	**.005**	**<.001**	**<.001**
C20:0	0^b^	0.2 ± 0.0^a^	0^b^	0.2 ± 0.0^a^	.079	**<.001**	.079
C20:1	0^c^	0.9 ± 0.0^a^	0^c^	0.8 ± 0.0^b^	**.029**	**<.001**	**.029**

*Mean ± standard error and significance (*p*) from a two‐way ANOVA (loin category, Cat; company, Com) with interaction. **Only those required by the current regulation (Orden PRE/3844/2004, n.d.). Different letters in the same row indicate significant differences (Tukey's test: *p* < .05). In bold: *p* ≤ .05.

**TABLE 2:** Results* for the microbial counts (log CFU g^−1^), instrumental color, and lipid and protein oxidation of vacuum‐packaged sliced loin from two categories and two companies without (Control) or with HPP before storage.

	Company A				Company B				*p*					
	Bellota 100% Iberian		Cebo		Bellota 100% Iberian		Cebo		Factors			Interaction		
	Control	HPP	Control	HPP	Control	HPP	Control	HPP	Cat	Com	HPP	Com × Cat	Cat × HPP	Com × HPP
Mesophilic aerobic bacteria	5.8 ± 0.1^bc^	4.9 ± 0.0^e^	6.5 ± 0.2^a^	5.0 ± 0.1^de^	5.7 ± 0.2^cd^	4.9 ± 0.1^e^	6.5 ± 0.3^ab^	5.7 ± 0.1^cd^	**<.001**	.192	**<.001**	.098	.251	.084
Lactic acid bacteria	4.1 ± 0.2^bc^	3.2 ± 0.8^c^	6.1 ± 0.1^ab^	3.3 ± 0.8^c^	6.0 ± 0.3^ab^	4.5 ± 0.1^bc^	6.8 ± 0.1^a^	5.4 ± 0.3^ab^	**.010**	**<.001**	**<.001**	.790	.161	.493
Molds and yeasts	3.7 ± 0.2^a^	3.9 ± 0.1^a^	3.8 ± 0.0^a^	4.2 ± 0.1^a^	4.1 ± 0.2^a^	<1^d^	2.7 ± 0.3^b^	1.0 ± 0.1^c^	.992	**<.001**	**<.001**	.174	**<.001**	**<.001**
Coliforms	4.3 ± 0.3^ab^	3.0 ± 0.3^bcd^	4.7 ± 0.4^a^	3.2 ± 0.2^abc^	1.8 ± 0.4^cd^	1.5 ± 0.6^d^	3.8 ± 0.2^ab^	1.4 ± 0.3^d^	**.020**	**<.001**	**<.001**	.267	**.041**	.969
*E. coli*	1.6 ± 0.4^a^	<1b	1.6 ± 0.5^a^	<1^b^	<1^b^	<1^b^	<1^b^	<1^b^	.973	**<.001**	**<.001**	.973	.973	**<.001**
L*	43.4 ± 0.2^ab^	44.5 ± 0.3^ab^	44.1 ± 0.4^ab^	46.3 ± 1.6^a^	42.9 ± 0.4^ab^	42.5 ± 0.8^b^	45.2 ± 0.6^ab^	45.5 ± 1.1^ab^	**.002**	.319	.155	.254	.428	.132
a*	8.2 ± 0.2^abc^	9.0 ± 0.3^a^	8.2 ± 0.5^abc^	8.5 ± 0.5^ab^	6.6 ± 0.3^bcd^	6.4 ± 0.7^cd^	4.2 ± 0.3^d^	4.8 ± 0.2^cd^	**<.001**	**<.001**	.233	**.005**	.794	.498
b*	4.1 ± 0.2	4.9 ± 0.3	4.7 ± 0.5	5.8 ± 1.0	3.3 ± 0.2	3.6 ± 0.8	4.7 ± 0.4	4.9 ± 0.7	**.011**	.064	.150	.455	.867	.411
C*	9.2 ± 0.2^ab^	10.2 ± 0.4^a^	9.5 ± 0.7^ab^	10.4 ± 1.0^a^	7.4 ± 0.3^bc^	7.4 ± 1.0^bc^	6.3 ± 0.4^c^	6.9 ± 0.4^bc^	.517	**<.001**	.154	.274	.775	.416
*H*°	26.7 ± 0.7^b^	28.2 ± 0.9^b^	29.6 ± 1.1^b^	33.2 ± 3.3^b^	26.5 ± 1.7^b^	28.0 ± 2.8^b^	47.9 ± 2.2^a^	44.8 ± 4.4^a^	**<.001**	**<.001**	.617	**<.001**	.708	.347
MDA (mg kg^−1^)	1.1 ± 0.0^a^	0.9 ± 0.1^a^	1.0 ± 0.1^a^	0.9 ± 0.0^a^	0.4 ± 0.0^b^	0.4 ± 0.0^b^	0.4 ± 0.0^b^	0.4 ± 0.1^b^	.193	**<.001**	.089	.144	.196	.140
Carbonyls (Nmol mg^−1^ protein)	3.4 ± 0.1^abcd^	3.0 ± 0.1^bcd^	2.5 ± 0.1^d^	2.6 ± 0.1^cd^	4.0 ± 0.3abc	4.5 ± 0.6a	4.1 ± 0.2^ab^	4.6 ± 0.5^a^	.261	**<.001**	.398	.074	.564	.213

*Note*: Different letters in the same row indicate significant differences (Tukey's test: *p* < .05). In bold: *p* ≤ .05.

* Mean ± standard error and significance (*p*) from a three‐way ANOVA (loin category, Cat; company, Com; and high‐pressure processing, HPP) with interaction.

With respect to the chemical composition, the Bellota 100% Iberian loins showed a lower moisture content than the Cebo (50% Iberian × Duroc) ones. This can be the result of the different ripening duration (companies usually set longer durations for the highest grade loins, in this case, approx. 85 vs. 70 days) and the IMF content. Differences in the IMF content (Table 1) can be explained in terms of differences in genetics (Ramirez & Cava, 2007) and pig management (Mayoral et al., 1999; Ortiz et al., 2021). It should be noted that IMF also depends on whether there is a selection of fat‐rich raw loins prior to processing, which is customary in some companies. All those factors vary according to each company's procedures, being usually adjusted to meet the consumers' expectations for densest marbling in the best quality Iberian products. In this regard, differences in IMF between companies were significant (*p* = .015), with an effect comparable to that of the loin type (Table 1). The significant effect of the category and company on some fatty acids (18:2, 18:3, and 20:1 and 16:1, 18:3. 20:0, and 20:1, respectively) (Table 1) can be mainly attributed to differences in the pig diet (acorns and grass vs. concentrate feeding), and to a lesser extent in genetics (pure Iberian breed vs. at least 50% Iberian × Duroc) (Carrapiso et al., 2003, 2020).

Regarding the microbial counts for the pathogens, *S. aureus* and *C. perfringens* were always below the detection limits, and *Salmonella* spp. and *L. monocytogenes* were not detected in 25 g of sample (not shown in Tables). With respect to the other microbial groups, some counts were above the advisable values (e.g., *E. coli* in company A) (Table 2). The loin packages had been randomly taken from those produced for consumers, and those high counts reveal that microbial risk is a real safety concern, the implementation of additional measures to lower them to acceptable levels is required. Regarding the loin category, the effect was significant on the mesophilic bacteria, LAB, and coliforms. The lower category (Cebo loin) showed the higher counts (Table 2), which might have been favored by the higher moisture content (Table 1). The company had a greater influence on the microbial counts than the category itself, affecting all the microbial groups except the mesophilic bacteria. The results reveal that both the loin category and the company's procedures might have a noticeable repercussion on most microbial counts, and therefore, on the concerns for food safety and shelf life.

Regarding the instrumental color, Table 2 shows that all the coordinates except C* were different between the loins from the two categories. The differences between companies were comparable, with three color coordinates being significantly different (Table 2). These differences between loin types and companies might be mainly related to differences in the raw pork (e.g., related pig management, procedures for pork selection, and pork management) as well as the selection of seasonings, additives, and some processing parameters, which are key for color development. In this regard, the great influence of the company might explain the lack of consistency in the results from previous studies on Iberian loin color (Contador et al., 2021; Soto et al., 2008), and confirms that the instrumental color is not intrinsically linked to the loin category, nor is it a precise estimator of it.

In the case of the oxidative status, neither lipid nor protein oxidation was affected by the loin category. The lack of effect on lipid oxidation (Table 2) matches previous results by Contador et al. (2021). However, Ventanas, Estevez, et al. (2006) did not report consistent results and suggested that the TBARS method might not be suitable to evaluate the feeding system and genetics in Iberian loin, despite its common use in dry‐cured products. The variability of those data explained by the ANOVA (*R* ^*2*^ = .874) does not confirm a lack of appropriateness and, in fact, the method was suitable to reveal a significant effect of the company (Table 2). This indicates that company procedures (e.g., the choice of seasonings and packaging materials) have a larger influence on lipid oxidation than the category itself. With respect to protein oxidation, the lack of effect of the loin category (Table 2) does not match previous results reporting higher values in the lower category (Cebo loin) (Contador et al., 2021; Ventanas, Estevez, et al., 2006). It should be noted that oxidative stability is the result of the balance between pro‐oxidant and antioxidant factors (Cava et al., 2000). Such factors depend not only on the loin category (depending on rearing system and crossbreeding) but also on the processing (e.g., seasonings) and pig diet. In this regard, the diet composition for Cebo (indoor‐reared) pigs can be adjusted by modifying the concentrate ingredients, and that for Bellota (outdoor‐reared without concentrates) pigs undergo slight fluctuations with the annual availability of grass and acorns (Tejerina et al., 2011). As a result, a lack of consistency between studies regarding the effect of loin category on oxidation might be expected.

The results suggest that additional strategies might be advisable to ensure loin safety, and reveal marked differences between categories. However, due to the intracategory variations, it might not be feasible to use the variables in Tables 1 and 2 as category indicators. In addition, the diversity in the samples provides the opportunity to check if the beneficial and detrimental effects of HPP and storage temperature depend on the loin characteristics.

### 3.2 Effect of HPP on the loins measured before storage

The effect of HPP on the microorganisms before storage was marked (all microbial counts were affected, with a general decrease), whereas no effect on the instrumental color or oxidation variables was found (Table 2).

Regarding the microorganisms, the decrease in the mesophilic counts for the four loin groups (Table 2) is in line with previous results for sliced dry‐cured loin (Campus et al., 2008) and Iberian “salchichón” (Cava et al., 2021), although for thickly sliced (2 cm instead of the usual 1–2 mm) Cebo loin (Cava et al., 2021), no effect was reported. Similar inconsistencies in the effect of HPP have been reported in Iberian “salchichón”, with both a decrease and no effect on “salchichón” from two companies (Ramírez et al., 2022), and in Iberian chorizo, with a decrease in sliced chorizo (Carrapiso et al., 2023), but also both a decrease in half pieces and an increase in sliced chorizo (Martin et al., 2021; Trejo et al., 2021). This inconsistency indicates that the effect of HPP may depend on some product characteristics, such as thickness, and the initial counts (Carrapiso et al., 2023). Our results do not reveal any influence of the loin category or company on the HPP effect, both interactions being not significant (*p* = .251 and .084, respectively). Similar to the mesophilic bacteria, the LAB counts also underwent a drop after the HPP, without significant interaction between HPP and loin category or company (*p* = .161 and *p* = .493, respectively). Although the lack of interaction suggests that the effect of HPP on both microbial groups is not largely determined by those factors, a modest effect cannot be completely dismissed due to the relatively low *p*‐values for some of those interactions.

Regarding molds and yeasts, HPP caused a different trend depending on the company, with HPP × company interaction being noticeable (*p* < .001). Company B's samples showed a marked drop in counts (Table 2), in line with previous results for Cebo loins (Cava et al., 2021). In contrast, company A's samples did not show any decrease, probably because of the competitive advantage of having lower initial counts in other microorganisms (e.g., LAB), and by its lower water content, since low *a*_*w*_ hinders HPP in the inactivation of microorganisms (Hugas et al., 2002).

With respect to the coliforms, the significant drop caused by HPP was larger in the Cebo samples (HPP × category interaction: *p* = .041), which were the ones with the higher initial counts and moisture content. The higher moisture might have contributed to the larger decrease in the Cebo samples, as mentioned before. Therefore, the results indicate that, as long as the lower category loins have higher coliform counts and moisture (which is likely to occur in the points of sale), the effect of HPP will be more effective on them. HPP also caused a drop in *E. coli* in the samples where they had initially appeared (company A's samples), lowering them below the detection limits (Table 2).

The HPP treatment did not affect the instrumental color of the sliced loin (Table 2). This partially matches previous results on Cebo loins reporting a limited effect, only significant on a* (Cava et al., 2009) or L* (Cava et al., 2021). It also matches the results found on the instrumental color, reporting no effect for Iberian chorizo (Carrapiso et al., 2023; Cava et al., 2020) and for Iberian salchichón, reporting no effect (Cava et al., 2021) or a slight effect (Ramírez et al., 2022). Conversely, a strong effect of HPP affecting both L* and a* was reported in dry‐cured non‐Iberian loin (Campus et al., 2008).

The low susceptibility of Iberian products to HPP‐induced discoloration might be related to factors mitigating the detrimental effect of HPP, such as low moisture content and high antioxidant content. Moisture is involved in the pressure‐induced disruption of protein inter‐ and intramolecular forces (Bak et al., 2019), and the generally high antioxidant content in the Iberian products has already been suggested as a protecting factor against HPP‐induced discoloration and oxidation (Amaro‐Blanco et al., 2018). The lack of discoloration (Table 2) indicates that, just after production, the effect of HPP will not affect consumers' decisions based on loin appearance. In addition, the lack of interaction between HPP and loin category and company (Table 2) indicates that the HPP treatment (600 MPa for 8 min) has a similar negligible effect on color irrespective of the loin characteristics.

With respect to oxidation, the HPP treatment did not affect either lipid (*p* = .089) or protein oxidation (*p* = .398). A significant effect on lipid oxidation but not on protein oxidation before storage was reported on thickly sliced Cebo loin with a similar HPP treatment (Cava et al., 2021), and a significant effect on both lipid and protein oxidation was reported after applying milder pressures (200 and 300 MPa) (Cava et al., 2009). As mentioned for the instrumental color, the lack of consistency between studies might be due to further factors modulating the effect of HPP. It should be noted that both oxidation variables were markedly influenced by the manufacturing company, which suggests that any potential HPP‐induced change in oxidation could be negligible from a practical point of view.

In summary, the 600 MPa‐8 min processing caused, just after application, a general decrease in all the microbial counts, whose extent was influenced by loin category and/or company for molds and yeasts, coliforms and *E. coli*. The effect on the instrumental color and oxidation was not significant irrespectively of the loin category or the manufacturing company. This implies that most consumers might not perceive any quality loss in recently HPP‐treated sliced loin regardless of the Iberian loin characteristics.

### 3.3 Effect of loin category and company after 90‐day storage

The differences between the two loin types on the microbial counts diminished slightly after 90‐day storage (the mesophilic counts were no longer affected), whereas it increased on the color coordinates and the protein oxidation (Table 3). Similarly, the effect of the company was softer on the microorganisms, slightly stronger on the instrumental color, and remained significant on lipid and protein oxidation (Table 3).

**TABLE 3:** Significance from a four‐way ANOVA with interaction performed on the data for the microbial counts, instrumental color, and lipid and protein oxidation from vacuum‐packaged sliced loin.

	Factors				Interaction					
	Cat	Com	HPP	*T*	Cat × Com	HPP × Cat	HPP × Com	HPP × *T*	*T* × Cat	*T* × Com
Mesophilic aerobic bacteria	.480	.078	.123	.506	.058	.382	**.044**	.479	**.009**	**.004**
Lactic acid bacteria	**<.001**	**<.001**	**.002**	**<.001**	.383	.082	**.003**	.155	**<.001**	**.014**
Molds and yeasts	.354	**<.001**	**<.001**	**<.001**	**<.001**	.982	**<.001**	**.003**	.090	**.005**
Coliforms	**.045**	.712	.073	**<.001**	**.003**	.226	.141	**<.001**	**.038**	.385
*E. coli*	.417	.258	**.042**	.790	.236	.790	.954	.417	.750	**.026**
L*	**<.001**	.087	**<.001**	.188	**.011**	.076	.212	.327	.073	.681
a*	**.001**	**<.001**	.317	.099	**<.001**	**.015**	**.017**	.773	**.005**	**.001**
b*	**<.001**	**<.001**	**.001**	.344	.060	.585	.545	.377	.876	**.006**
C*	**<.001**	**<.001**	**.016**	.160	**<.001**	.096	.093	.777	.071	**.001**
*H*°	**.001**	**<.001**	**<.001**	.987	.796	**.013**	**.017**	.144	**.018**	.431
MDA	.082	**<.001**	**.012**	**.027**	**<.001**	**.001**	.279	**.004**	.642	.070
Carbonyls	**.035**	**<.001**	**<.001**	.122	.411	.636	.111	**.004**	**.002**	.300

*Note*: In bold: *p* ≤ .05.

Abbreviations: Cat, category; Com, company; HPP, high‐pressure processing; T, storage temperature.

Regarding the microbial counts, the initial differences between the loin types in the mesophilic bacteria disappeared after 90‐day storage, whereas they persisted in the LAB and coliforms (Table 3). Specifically, the LAB counts were still higher in the Cebo samples, but the coliforms showed an inconsistent trend (Table 4). Regarding the company, its effect still persisted in the LAB and molds and yeasts (Table 3). However, the initially significant effect on coliforms and *E. coli* was canceled out after storage, with *E. coli* counts below the detection limits in all the groups (Table 4). Therefore, storage diminished the initial differences related to loin category and company. These results partially match those from a previous study on Iberian “salchichón”, which reported that most of the initial differences between products in the microbial counts still persist after 90‐day storage.

**TABLE 4:** Results (mean ± standard error) for the microbial counts (log CFU g^−1^), instrumental color, and lipid and protein oxidation of vacuum‐packaged sliced loin.

	Company A								Company B							
	Bellota 100% Iberian				Cebo				Bellota 100% Iberian				Cebo			
	4°C		20°C		4°C		20°C		4°C		20°C		4°C		20°C	
	Control	HPP	Control	HPP	Control	HPP	Control	HPP	Control	HPP	Control	HPP	Control	HPP	Control	HPP
Mesophilic aerobic bacteria	6.0 ± 0.1^abcde^	5.8 ± 0.1^bcde^	6.6 ± 0.1^abc^	6.9 ± 0.2^a^	5.5 ± 0.4^de^	6.7 ± 0.3^ab^	6.4 ± 0.2^abcd^	5.3 ± 0.4^e^	6.4 ± 0.2^abcd^	5.6 ± 0.1^cde^	5.5 ± 0.0^de^	5.8 ± 0.2^bcde^	6.9 ± 0.1^a^	5.6 ± 0.0^cde^	5.7 ± 0.0^bcde^	5.6 ± 0.0^cde^
Lactic acid bacteria	3.6 ± 0.2^ef^	3.1 ± 0.^4f^	2.9 ± 0.0^f^	2.9 ± 0.0^f^	5.0 ± 0.2^bcd^	5.9 ± 0.3^ab^	3.4 ± 0.3^ef^	2.9 ± 0.0^f^	4.8 ± 0.2^cd^	3.6 ± 0.1^ef^	3.5 ± 0.2^ef^	4.4 ± 0.3^de^	7.0 ± 0.1^a^	5.0 ± 0.2^bcd^	5.7 ± 0.2^bc^	4.3 ± 0.3^de^
Molds and yeasts	2.0 ± 0.2^bcd^	<1^e^	<1^e^	<1^e^	2.9 ± 0.3^b^	3.0 ± 0.3^b^	<1^e^	<1^e^	4.8 ± 0.3^a^	1.2 ± 0.3^cde^	2.6 ± 0.3^bc^	1.3 ± 0.4^cde^	3.1 ± 0.4^b^	<1^e^	2.5 ± 0.3^bc^	<1^e^
Coliforms	1.9 ± 0.8^bcd^	2.5 ± 0.2^bcd^	3.3 ± 0.1^abcd^	5.3 ± 0.1^a^	2.4 ± 0.7^bcd^	2.1 ± 0.5^bcd^	1.1 ± 0.7^d^	2.2 ± 0.7^bcd^	2.5 ± 0.4^bcd^	1.4 ± 0.2^cd^	2.1 ± 0.4^bcd^	3.7 ± 0.7^abc^	2.6 ± 0.3^bcd^	1.3 ± 0.3^cd^	2.8 ± 0.5^bcd^	3.9 ± 0.1^ab^
*E. coli*	<1	<1	<1	<1	<1	<1	<1	<1	<1	<1	<1	<1	<1	<1	<1	<1
L*	43.7 ± 0.5^bcde^	43.3 ± 0.3^cde^	42.9 ± 0.2^def^	43.3 ± 0.3^cdef^	43.2 ± 0.3^cdef^	47.2 ± 1.0^a^	43.8 ± 0.4^bcde^	46.4 ± 0.6^ab^	42.0 ± 0.7^ef^	43.4 ± 0.5^cde^	40.4 ± 0.4^f^	41.9 ± 0.7^ef^	44.5 ± 0.2^abcde^	45.9 ± 0.7^abc^	46.0 ± 0.6^abc^	45.3 ± 0.9^abcd^
a*	9.1 ± 0.2^a^	8.8 ± 0.1^ab^	7.9 ± 0.3^abc^	8.5 ± 0.4^ab^	7.6 ± 0.4^abc^	9.1 ± 0.6^a^	7.8 ± 0.3^abc^	8.9 ± 0.2^ab^	4.1 ± 0.5^e^	3.9 ± 0.5^e^	4.8 ± 0.7^de^	3.5 ± 0.3^e^	4.9 ± 0.2^de^	4.6 ± 0.3^de^	6.3 ± 0.4^cd^	7.0 ± 0.5^bc^
b*	5.2 ± 0.2^abcd^	4.9 ± 0.2^bcd^	4.6 ± 0.3^bcd^	4.8 ± 0.3^bcd^	5.0 ± 0.4^abcd^	7.2 ± 0.7^a^	4.6 ± 0.3^bcd^	6.5 ± 0.3^ab^	2.0 ± 0.5^e^	4.2 ± 0.5^cd^	3.4 ± 0.6^de^	4.2 ± 0.5^cd^	4.7 ± 0.2^bcd^	4.8 ± 0.3^bcd^	6.0 ± 0.4^abc^	5.7 ± 0.7^abc^
C*	10.4 ± 0.2^ab^	10.1 ± 0.2^ab^	9.2 ± 0.4^abc^	9.8 ± 0.5^ab^	9.2 ± 0.5^abc^	11.6 ± 0.9^a^	9.1 ± 0.4^abc^	11.0 ± 0.4^ab^	4.6 ± 0.6^d^	5.7 ± 0.7^d^	5.9 ± 0.9^d^	5.5 ± 0.5^d^	6.8 ± 0.3^cd^	6.7 ± 0.3^cd^	8.7 ± 0.3^bc^	9.0 ± 0.8^abc^
*H*°	29.7 ± 1.0^ef^	29.3 ± 0.6^ef^	30.1 ± 1.0^ef^	29.3 ± 0.5^ef^	33.4 ± 1.4^def^	37.9 ± 1.9^bcde^	30.5 ± 0.8^ef^	36.2 ± 0.8^cde^	24.7 ± 4.8^f^	47.0 ± 2.0^ab^	34.9 ± 1.9^def^	48.7 ± 3.0^a^	43.6 ± 1.2^abcd^	46.5 ± 2.8^abc^	43.4 ± 2.8^abcd^	38.8 ± 2.1^abcde^
MDA (mg kg^−1^)	1.2 ± 0.1^ab^	1.1 ± 0.0^abc^	0.9 ± 0.0^cd^	1.1 ± 0.0^ab^	1.3 ± 0.0^a^	1.1 ± 0.0^ab^	1.1 ± 0.0^bc^	1.2 ± 0.0^ab^	0.5 ± 0.1^ef^	0.8 ± 0.0^d^	0.5 ± 0.0^ef^	0.7 ± 0.1^de^	0.4 ± 0.0^f^	0.4 ± 0.0^f^	0.5 ± 0.1^f^	0.4 ± 0.0^f^
Carbonyls (Nmol mg^−1^ protein)	4.0 ± 0.2^cdef^	3.9 ± 0.1^cdef^	3.2 ± 0.1^ef^	4.4 ± 0.^3cdef^	3.5 ± 0.1^def^	3.5 ± 0.2^def^	3.1 ± 0.1^f^	4.6 ± 0.2^cdef^	5.6 ± 0.6^abc^	6.4 ± 0.6^ab^	4.9 ± 0.5^bcde^	6.4 ± 0.3^ab^	4.2 ± 0.7^cdef^	5.1 ± 0.2^abcd^	5.2 ± 0.3^abcd^	6.8 ± 0.2^a^

*Note*: Different letters in the same row indicate significant differences (Tukey's test: *p* < .05).

Regarding the instrumental color, the marked initial effect of loin category and company increased slightly after storage, with additional differences appearing in C* and b*, respectively. Results match those by Contador et al. (2021), who reported a similar trend in the effect of the Iberian loin category before and after storage, and those by Ramírez et al. (2022) for Iberian “salchichón”. It should be noted that the effect of the company was still comparable to that of the loin category, with *p* < .001 for four of the coordinates (Table 3).

With respect to the oxidative status, the initial lack of effect of the loin category on lipid oxidation persisted after storage, whereas differences in protein oxidation did appear, with the Bellota 100% Iberian loins having the higher values. This suggests that initial prooxidant or antioxidant factors might persist throughout storage, modulating the development of the oxidation reactions. The company still affected lipid oxidation (values for company A nearly doubled those of company B) and protein oxidation (company B reached the highest values in all the groups) (Table 4). These results partially match those by Contador et al. (2021), who reported no differences between the two loin categories in the lipid and protein oxidation after 4‐month storage, and those by Ramírez et al. (2022), who reported that the initial differences between two types of “salchichón” persisted for lipid oxidation, but not for protein oxidation after 90‐day storage.

The company still had a stronger effect on oxidation than the loin category (Table 3).

### 3.4 Effect of HPP measured after 90 days

After storage, the initially significant effect of HPP on all the microorganism counts diminished, whereas a detrimental effect appeared on the instrumental color (all the coordinates except a*) and lipid and protein oxidation (Table 3).

Regarding the microbial counts, the effect of HPP was softened by storage (Table 3). The mesophilic counts and coliforms were no longer affected, and the initially marked decrease in LAB (Table 2) was replaced by an inconsistent effect (Table 4). A general decrease in the effectiveness of HPP in reducing the microbial counts after storage was also reported in Cebo loin, “salchichón” (Cava et al., 2021) and chorizo (Carrapiso et al., 2023; Cava et al., 2020; Trejo et al., 2021). Conversely, the initial drop in molds and yeasts and *E. coli* (Table 2) persisted (Tables 3 and 4). The lack of effect on the mesophilic counts can be a result of their recovery to nearly the pre‐HPP levels. For the LAB, the effect of HPP was lessened after storage as a consequence of the decrease in counts caused by the storage itself. In fact, storage caused a decrease even larger than the HPP treatment itself. Regarding the molds and yeasts, the effect of HPP persisted after 90 days, with a general decrease to near or below the detection limits.

The instrumental color, not influenced by HPP before storage, was greatly affected by HPP after 90 days (all the coordinates except a*, Table 3), with a general increase in L*, b*, and C*. The results are in line with those reported in Cebo loins with a similar HPP treatment (Cava et al., 2021), although at milder HPP conditions (200 and 300 MPa for 15 or 30 min) no effect of HPP was found after storage for 60 and 90 days (Cava et al., 2009). In addition, the effect of HPP was not influenced by loin category or company. The results indicate that the HPP‐induced reactions still develop during storage, yielding a noticeable discoloration that will affect consumers' decisions more likely as storage time increases.

Regarding oxidation, HPP had an effect similar to that on the instrumental color, the harmful HPP‐induced reactions taking place mainly over time instead of in the short term. The effect of HPP on lipid oxidation was influenced by the loin category, with a significant interaction (*p* < .001) (Table 3). In this regard, HPP caused a general increase in the MDA content in the Bellota 100% Iberian loins but had no effect on the Cebo ones (Table 4). This suggests that the detrimental effect of HPP might depend on the loin characteristics. In addition, the effect of HPP was also affected by the storage temperature (interaction: *p* = .004), HPP resulting in a slightly larger increase in lipid oxidation at 20°C than at 4°C (Table 4). HPP resulted in a general rise in protein oxidation (Table 4) regardless of the loin category and company (Table 3). Similar to lipid oxidation, the increase in the HPP‐treated samples was slightly larger after storage at 20°C than at 4°C, with a significant HPP–temperature interaction (*p* = .004). Previous results on sliced Cebo loin have reported inconsistent results (Cava et al., 2021; Trejo et al., 2021), again probably because of additional factors affecting the results.

In summary, the 90‐day storage caused a decrease in the beneficial effect of HPP to reduce the microbial counts (e.g., the effect of HPP on the coliforms was lost after storage) and an increase in its detrimental effect on color and oxidation. This suggests that, in terms of both food safety and quality, HPP might be more convenient when storage is limited, and that extended storage might soften the beneficial effect on microorganisms to the extent of making it insufficient to outweigh the increasingly harmful effect on color and oxidation. Therefore, HPP can increase loin safety in the short term, causing in turn a decrease in the shelf‐life.

### 3.5 Effect of storage temperature

The effect of the storage temperature (4°C vs. 20°C) was significant on the LAB, molds and yeasts, coliforms, and lipid oxidation, whereas no effect was found on the mesophilic bacteria, the instrumental color and protein oxidation (Table 3).

LAB and molds and yeasts consistently had lower counts after storage at 20°C than at 4°C, whereas no differences appeared in the mesophilic bacteria counts (Table 3). These results partially match previous results, which reported lower counts for the mesophilic bacteria and molds and yeast using 20°C than at 4°C for 120‐day storage in Cebo loin (Cava et al., 2021). Although there is no previous information about the effect of the storage temperature on the LAB in loin, the results (Tables 3 and 4) are generally in line with those reported in chorizo, with lower counts after storage at 20°C than at 4°C (Carrapiso et al., 2023; Martin et al., 2021; Trejo et al., 2021). The lower counts at 20°C than at 4°C in those microorganisms also matches the results for the *L. monocytogenes* counts in Cebo loin (Cava et al., 2021) and other dry‐cured Iberian products (Cava et al., 2020, 2021).

However, it should be noted that the coliform counts were generally higher after storage at 20°C than at 4°C (Table 4). The growth of coliforms during storage at 20°C might have been favored by the drop in other microbial groups, such as LAB and molds and yeasts, and the decreased competition for resources enabling coliforms to thrive. The effect of the storage temperature depended significantly on the HPP treatment and the loin category (interaction: *p* < .001 and .038, respectively, Table 3). In this regard, storage at 20°C resulted in higher coliform counts in the HPP‐treated samples than in the Control ones in all the cases (Table 4), although only the HPP Bellota samples stored at 20°C greatly exceeded the initial counts (Table 2). In addition, 20°C storage caused a general increase in the counts of the Bellota 100% Iberian loins, but a general decrease in coliforms in the Cebo loins irrespectively of the temperature (compare Tables 2 and 4). Therefore, 20°C for 90‐day storage was not suitable to control the coliform counts in every Iberian loin group, which suggests that those storage conditions should not be applied to Iberian loin during commercialization unless no safety issues related to growth coliform have been proven for the specific characteristics of the Iberian loin produced. The effect of storage temperature had never been reported in this product to our knowledge. Recent studies on Iberian “salchichón” (Ramírez et al., 2022) and chorizo (Carrapiso et al., 2023) reported no effect or a decrease in the coliform counts, which match some of our results for some loin groups, but not for others, which may be due to the differences in the initial characteristics. Therefore, despite the beneficial effect of 20°C to decrease some microbial counts, the results for the coliforms challenge the application of such temperatures during long storage. The results also question the use of HPP to ensure loin safety when refrigeration during long‐term distribution and storage cannot be adequately maintained, and highlight the need for low initial coliform counts and refrigeration during long‐term storage.

With respect to the instrumental color, the effect of the storage temperature was not significant. Results are in line with a previous study on Cebo loin reporting a marginal effect, only significant for L* after 120 days (Cava et al., 2021), and with the lack of effect on sliced chorizo (Carrapiso et al., 2023; Martin et al., 2021; Trejo et al., 2021). Regarding oxidation, the storage temperature had a significant (*p* = .027) yet inconsistent effect on lipid oxidation regardless of the loin category and company, and no effect on protein oxidation (Table 3). The results agree with a previous study on Cebo Iberian loin, which showed a slight effect of the storage temperature on lipid oxidation (significant on day 30, but not on days 60 or 120) and no effect on protein oxidation (Cava et al., 2021). Previous studies on dry‐cured Iberian chorizo have reported an inconsistent effect of the storage temperature on lipid and protein oxidation.

Regarding lipid oxidation after 90 days, no effect of the storage temperature (Carrapiso et al., 2023; Martin et al., 2021; Trejo et al., 2021), but also more lipid oxidation at 4°C than at 20°C (Ramírez et al., 2022) was reported. With respect to protein oxidation, inconsistent results after 90 days have been reported: no differences (Ramírez et al., 2022), but also higher values at 4°C than at 20°C (Carrapiso et al., 2023), and opposite trend (Martin et al., 2021; Trejo et al., 2021). These inconsistencies might be related to additional factors, different in each study. Among them, the packaging materials and their oxygen permeability might have been involved in the differences between studies. Further studies are advisable on the effect of these materials on the oxidative changes, as well as on the microbial counts, during storage at different temperatures.

In summary, storage at 20°C resulted in generally lower microbial counts than at 4°C and a minimal effect on the instrumental color and oxidation. However, the trend for coliforms was the opposite, which raises concerns about loin safety stored at 20°C.

## 4 CONCLUSIONS

The differences in composition, microbial counts, instrumental color, and oxidative stability between Iberian loins from different categories are comparable to those between two leading manufacturers. This company's variability might explain some of the lack of consistency among previous studies on Iberian loin. In addition, both the loin category and company modulate the effect of the HPP treatment on some microbial counts before storage, and as well the company does after storage. Both also modulate the effect of the storage temperature. Regarding the HPP treatment, results indicate that storage decreases its advantages, softening the initial HPP‐induced microbial drop and allowing the development of a harmful effect of HPP on the instrumental color and oxidation. This suggests that storage duration should be limited for HPP‐treated loin to obtain the most beneficial effect. Further research on the improvement of packaging materials are advisable to lessen the quality damage induced by HPP during the subsequent storage. Finally, results shed light on a not yet researched safety issue, which is the effect of the storage temperature on the coliform counts of Iberian loin, revealing that 20°C is less advisable than 4°C for 90‐day stored sliced Iberian loin. Contrary to results from previous studies on *Listeria* spp., 20°C storage did not result in a drop in the coliform counts. This highlights the requirement for low initial counts since HPP and 20°C storage might not be sufficient to ensure food safety in stored loin.

## AUTHOR CONTRIBUTIONS

**A.I. Carrapiso:** Conceptualization (equal); formal analysis (equal); writing – original draft (lead); writing – review and editing (equal). **David Tejerina:** Methodology (equal); writing – review and editing (equal). **Susana García‐Torres:** Methodology (equal); writing – review and editing (equal). **Antonia Trejo:** Data curation (equal); methodology (equal). **Maria Jesus Martin Mateos:** Data curation (equal); formal analysis (equal). **Jonathan Delgado‐Adamez:** Methodology (equal); writing – review and editing (equal). **J.J. García‐Parra:** Methodology (equal); supervision (equal); writing – review and editing (equal). **Rosario Ramírez:** Conceptualization (equal); project administration (lead); supervision (equal).

## FUNDING INFORMATION

This work was supported by the INNOACE project [Interreg, European Regional Development Fund (ERDF)] and also by MEAT Project (European Regional Development Fund).

## CONFLICT OF INTEREST STATEMENT

The authors declare no conflict of interest.

## ETHICS STATEMENT

Ethical review and approval were waived for this study due to the use of standard procedures of sensory analysis and samples commercially available. Informed consent was obtained from all subjects involved in the study.

## Data Availability

The data that support the findings of this study are available on request from the corresponding author.
